# Case Report: Overlap of long QT syndrome and catecholaminergic polymorphic ventricular tachycardia in two Chinese children with *CALM2*-related calmodulinopathy

**DOI:** 10.3389/fped.2026.1902628

**Published:** 2026-07-22

**Authors:** Jinzhi Wu, Jianguang Qi, Ying Liao

**Affiliations:** 1 Department of Pediatrics, Children’s Medical Center, Peking University First Hospital, Beijing, China; 2 Department of Pediatrics, Kaili First People’s Hospital, Kaili, China

**Keywords:** *CALM2*, calmodulinopathy, case report, catecholaminergic polymorphic ventricular tachycardia, long QT syndrome, overlap phenotype

## Abstract

**Introduction:**

Calmodulinopathy is a rare *CALM*-related hereditary channelopathy presenting with long QT syndrome (LQTS), less commonly with catecholaminergic polymorphic ventricular tachycardia (CPVT) or LQTS/CPVT overlap. It carries high mortality and limited data from Chinese population.

**Methods:**

We reviewed two Chinese children with *CALM2* mutation-related LQTS/CPVT overlap. Clinical data, genetic findings, and treatments were collected; outcomes were obtained via telephone follow-up. A systematic literature review was also performed.

**Results:**

Case 1 was a 13-year-old girl with seven exercise-induced syncopal episodes. Sinus rhythm QTc 0.55 s, with bidirectional/polymorphic premature ventricular contractions, and ventricular tachycardia during exercise. A *de novo CALM2* p.E140Q mutation was found. She remained event-free on propranolol and propafenone for two years. Case 2 was a 15-year-old boy with onset at 4 years. He experienced > 10 syncopal episodes triggered by recurrent exercise or emotional stress. Initial Holter monitoring revealed sinus bradycardia with a mean heart rate of 70 bpm and a maximum QTc of 0.51 s. A *de novo CALM2* p.N98S mutation was found. Despite treatment with propranolol plus mexiletine, syncopal episodes continued, with the QTc interval further prolonged to 0.62 s. The p.E140Q variant is novel, whereas p.N98S has been previously reported. Literature suggests that β-blockers combined with Class Ic drugs may be more effective, and implantable cardioverter defibrillator (ICD) implantation should be considered in cases who respond poorly to medical therapy or survivors of aborted cardiac arrest. Patients who experience recurrent syncope coexisting with significant sinus bradycardia may have more severe phenotypes with higher risk of malignant arrhythmias, warranting earlier or more aggressive evaluation for device therapy.

**Conclusion:**

Genetic testing is recommended for suspected pediatric channelopathies with phenotype-guided individualized treatment.

## 1 Introduction

Calmodulin (CaM) is a ubiquitous calcium-binding messenger protein encoded by *CALM1*, *CALM2*, and *CALM3*. It regulates numerous ion channels and signaling pathways in cardiomyocytes (1). Since the first report in 2012 linking *CALM* mutations to ventricular tachycardia and sudden cardiac death, calmodulinopathy has been recognized as an important genetic etiology of malignant arrhythmias in children (2, 3). The clinical presentation of calmodulinopathy is heterogeneous, manifesting as long QT syndrome (LQTS), catecholaminergic polymorphic ventricular tachycardia (CPVT), an overlap phenotype in which both manifestations coexist (LQTS/CPVT), or idiopathic ventricular fibrillation. The overlap phenotype is characterized by concurrent QTc prolongation and catecholamine-induced bidirectional/polymorphic ventricular tachycardia. This entity is clinically rare and poses greater challenges in diagnosis and management (4). The article reports two independent Chinese pediatric cases of LQTS/CPVT overlap phenotype associated with *CALM2* gene mutations, and discusses their clinical characteristics, genetic basis, and treatment strategies in the context of a literature review.

## 2 Case presentation

### 2.1 Case 1

A 13-year-old Chinese girl presented with recurrent syncope within 15 months. She experienced a total of 7 episodes, all occurring during strenuous exercise (for example, fast running, fast cycling or frog jumps). The episodes were characterized by no prodrome, collapse with loss of consciousness, no convulsions, with or without urinary incontinence, lasting less than 10 min, although the exact durations were unknown. Any exercise-related chest pain was denied. Besides, she occasionally experienced palpitations and dizziness when climbing upstairs.

She was born at 36 weeks of gestation. The motor and mental development were normal. There was no family history of inherited cardiovascular disease, sudden cardiac death, or drowning.

**Figure 1:**
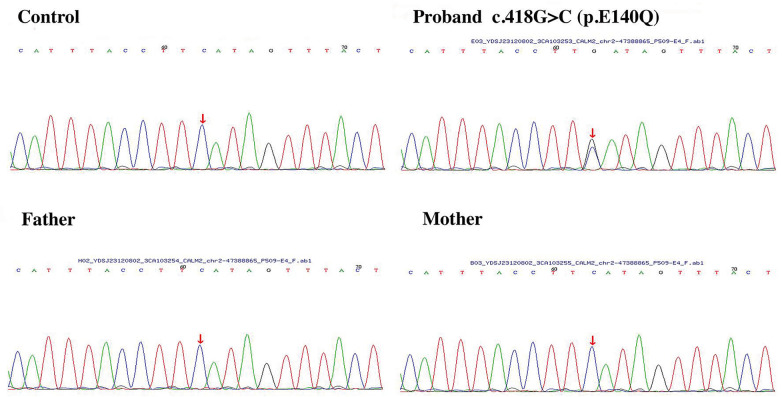
Nucleotide sequence analysis of case 1. The patient carried a *de novo CALM2* c.418G > C (p.E140Q) mutation, which was not present in either parent.

The physical examination revealed no abnormal findings. The blood routine tests, electrolytes, cardiac enzymes were within normal limits. Cranial magnetic resonance imaging, magnetic resonance angiography, and electroencephalography showed no abnormalities. 12-lead electrocardiogram (ECG) was essentially normal with a borderline QTc of 0.43 s. Echocardiography showed normal cardiac structure and function. Next-generation sequencing of peripheral blood DNA revealed a novel *de novo* *CALM2* variant of c.418G > C (p.E140Q), confirmed by parental Sanger sequencing (Figure 1).

**Figure 2:**
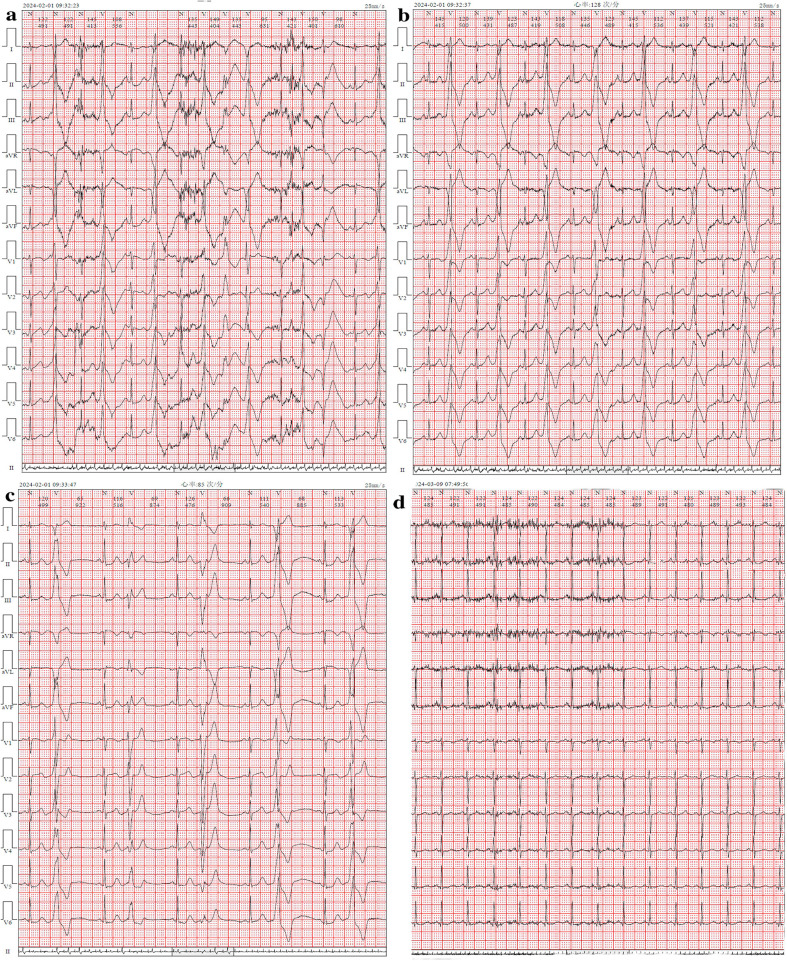
The Holter monitoring results of the exercise stress test for case 1. **(a)** During the exercise period, frequent polymorphic premature ventricular complexes were observed, including bigeminy, with some appearing in couplets and showing a bidirectional pattern. **(b)** During the exercise period, polymorphic premature ventricular complexes were observed at a heart rate of 128 bpm. **(c)** During the recovery period after exercise test cessation, polymorphic premature ventricular complexes appeared as the heart rate dropped to 85 bpm. **(d)** One month after treatment, Holter recording demonstrated the absence of premature ventricular complexes during exercise testing, with a heart rate of 123 bpm.

A diagnosis of possible LQTS was considered and the patient was first treated with propranolol at 0.5 mg/kg/day and restricted from exercise. Nevertheless, under the treatment of propranolol, her Holter ECG monitoring showed sinus rhythm with a mean heart rate of 59 beats per minute (bpm) and intermittent QTc prolongation (maximum QTc 0.55 s); meanwhile, notably, polymorphic premature ventricular contractions, including paired bidirectional premature ventricular beats and polymorphic non-sustained ventricular tachycardia can be observed when her heart rate exceeded 120 bpm during an exercise stress test (Figures 2a–c). Treatment was adjusted to propranolol at 0.7 mg/kg/day combined with propafenone at 275 mg/day in consideration of an LQTS/CPVT overlap phenotype related to *CALM2* variant, along with restriction from vigorous exercise. Implantable cardioverter defibrillator (ICD) implantation was recommended concurrently, but the family declined after detailed counseling. They expressed concerns about the long-term risks of the device in a young child, including the need for repeated generator replacements over her lifetime and the potential psychological burden. The parents also reported that the patient herself was fearful of the procedure. Accordingly, they preferred to continue pharmacological management with regular follow-up and reassessment of ICD need if symptoms recurred.

One month after treatment, repeat Holter monitoring showed a mean heart rate of 67 bpm; no premature ventricular beats were observed during exercise stress when the heart rate exceeded 120 bpm (Figure 2d), and the maximum QTc was 0.50 s. During the two-year follow-up, the patient experienced no recurrence of syncope and denied any symptoms (e.g., dizziness or nausea). Good adherence and tolerability were confirmed at each visit.

### 2.2 Case 2

A 15-year-old Chinese boy who presented with recurrent syncope over 11 years with an onset at 4 years old. All the episodes were triggered by strenuous exercise or emotional stress and were preceded by brief dizziness and blurred vision. He experienced loss of consciousness and pallor or cyanosis within a few minutes during the episodes, with or without urinary incontinence. No convulsions were observed. There were about 10 episodes within the first three years.

He was born full-term, with normal exercise tolerance and normal motor and mental development. His mother had a history of fainting during emotional excitement in childhood, and a maternal cousin had a history of syncope, although details were unclear. His two older sisters were healthy.

**Figure 3:**
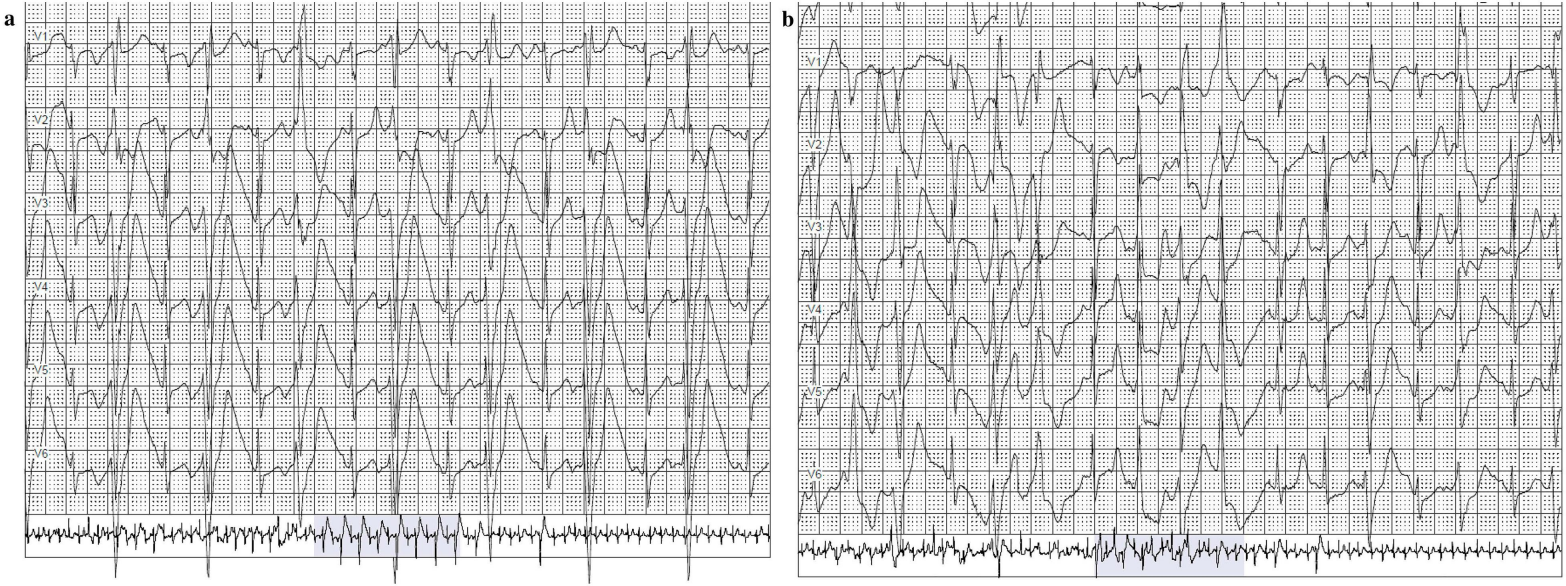
The Holter monitoring results of the exercise stress test for case 2. **(a, b)** During the Holter monitoring exercise test, polymorphic premature ventricular complexes were observed, with some showing a bidirectional pattern.

At age 7, the patient was hospitalized for evaluation. Bidirectional and polymorphic premature ventricular contractions were observed after exercise (Figure 3), with a maximum QTc of 0.51 s, which was recorded during Holter monitoring that also showed sinus bradycardia with a mean heart rate of 70 bpm. Echocardiography showed mild left ventricular enlargement and a false tendon in the left ventricle. Cranial magnetic resonance imaging, magnetic resonance angiography, and electroencephalography revealed no abnormalities. Treatment was initiated with propranolol at 1.9 mg/kg/day, and exercise was restricted.

At age 8, next-generation sequencing of peripheral blood DNA identified a *de novo* mutation in the *CALM2* gene, c.293A > G (p.N98S), confirmed by Sanger sequencing in the parents and two older sisters. A diagnosis of LQTS type 15 was made, and treatment with propranolol was continued. At age 9, repeat echocardiography still showed mild left ventricular enlargement and a false tendon, but all subsequent repeated examinations returned to normal.

At age 11, the local hospital reduced the propranolol dose to 0.02 mg/kg/day due to perceived adequate symptom control; however, the patient subsequently experienced two recurrences, and was again evaluated at our hospital. In view of the established diagnosis of LQTS, we decided to add mexiletine. Prior to initiating mexiletine, a lidocaine test performed and yielded a negative result, with QTc shortening from 0.454 s to 0.448 s (*Δ* = –0.006 s). The treatment regimen was then adjusted to a combination of propranolol (0.8–1.3 mg/kg/day) and mexiletine (6.3–8.1 mg/kg/day).

At age 14, one additional recurrence occurred. ICD implantation was recommended by the clinical team after thorough risk-benefit discussion. However, the family declined the procedure. They expressed concerns about the potential risks of device-related complications (e.g., inappropriate shocks, lead failure, and infection) and the psychological impact of living with an ICD on their teenage son. They also worried that the device might restrict his participation in sports and daily physical activities, which they considered essential for his quality of life. After detailed counseling, the family opted to continue pharmacological management with close follow-up, and ICD candidacy was to be reassessed if symptoms recurred.

At the final assessment at age 15, physical examination revealed bradycardia with a heart rate of 40 bpm and regular rhythm, and Holter monitoring showed sinus bradycardia with a maximum QTc of 0.62 s. An LQTS/CPVT overlap phenotype attributable to the *CALM2* mutation was considered. Treatment was initiated with the addition of propafenone, and mexiletine was reduced as appropriate. ICD implantation was recommended again (Table 1). Throughout the entire treatment course, the patient tolerated all medications well without any adverse effects.

**Table 1:** Timeline of key clinical events, interventions, and Holter monitoring changes in case 2.

Patient age	Age 7	Age 8	Age 9	Age 11	Age 11	Age 12	Age 13	Age 14	Age 15
Events	Syncope	Event-free	Event-free	Syncope	Event-free	Event-free	Event-free	Syncope	Event-free
propranolol (mg/kg/d)	Not receiving	1.9	1.9	0.02	1.3	1.3	0.9	0.8	0.6
mexiletine (mg/kg/d)	Not receiving	Not receiving	Not receiving	Not receiving	6.3	8.1	7.4	6.6	7.8
Mean heart rate (bpm)	70	62	69	50	55	52	53	53	44
Maximum QTc (s)	0.51	0.51	0.49	0.55	0.57	0.58	0.55	0.52	0.62
Exercise ECG	Polymorphic PVCs	Negative	Negative	QTc prolongation	Negative	Negative	Negative	Negative	Negative

ECG electrocardiogram; PVCs, premature ventricular contractions.

## 3 Discussion

We report two Chinese pediatric cases carrying novel *CALM2* mutations (p.E140Q and p.N98S). Both presenting with episodes of syncope triggered by exercise or emotional stress, prolonged QTc intervals and exercise-induced ventricular arrhythmias in ECG or Holter monitoring, consistent with the characteristics of the LQTS/CPVT overlap phenotype, which is one of the rare phenotypes of calmodulinopathy.

CaM mutations can interfere with calcium binding and downstream channel regulation. Depending on the mutation, this can lead to impaired calcium-dependent inactivation of L-type Ca_v_1.2 calcium channels (a key mechanism for LQTS) and/or dysfunctional regulation of RyR2 (central to CPVT), thereby producing LQTS and CPVT phenotypes, either individually or in combination (1, 4, 5).

**Figure 4:**
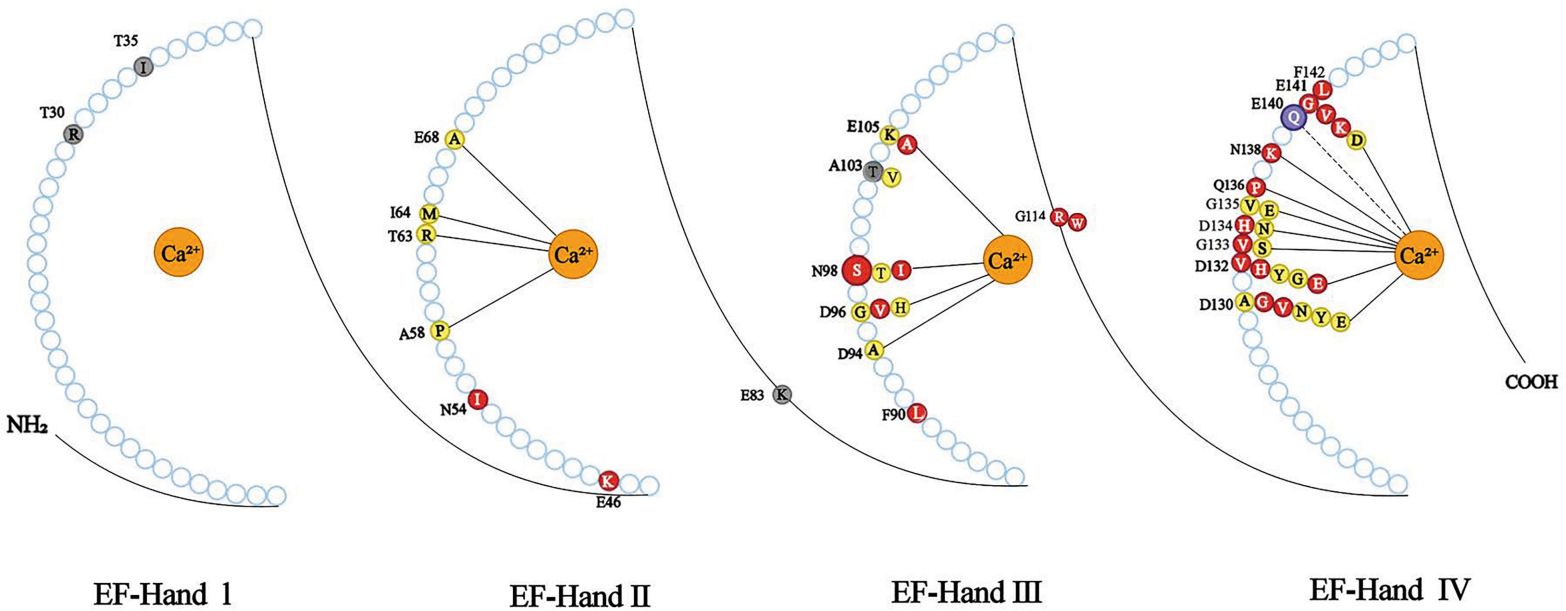
The relationship between variant location and phenotype in *CALM1-3*. The names of the original amino acids and their numbers are indicated near each symbol, and the mutated amino acids are represented by colored symbols. According to the American College of Medical Genetics and Genomics classification criteria, red indicates pathogenic, yellow indicates likely pathogenic, and gray indicates suspected variant of uncertain significance. The purple site represents case 1 in this study. The solid-line connection to calcium ions represents a mutation occurring in Ca^2^⁺ -chelation within the protein domain. p.E140, a novel site identified here and connected by a dashed line, suggests an unclear but highly suspected link to the same mutation.

In case 1, the mutation site has not been previously reported. The variant is classified as likely benign based on the protein function prediction software Rare Exome Variant Ensemble Learner version 1.3 (6); and according to the American College of Medical Genetics and Genomics guidelines, it was preliminarily classified as a variant of uncertain significance. However, several lines of evidence support its potential pathogenicity. First, p.E140Q is a *de novo* mutation located within EF-hand motif IV, a known hotspot for pathogenic mutations; second, mutations at the adjacent sites p.N138, p.E141 and p.F142 have all been confirmed as “pathogenic” in previous reports (Figure 4) (4, 7, 8); third, according to the CPVT clinical diagnostic scoring system (9, 10), the patient scored 3 points (1 point for symptoms, 2 points for the exercise stress test, and 0 points for all other items), corresponding to an intermediate pretest probability of CPVT (approximately 50% likelihood). Under the phenotype-enhanced American College of Medical Genetics and Genomics framework developed for *RYR2*, a CPVT clinical score of 2–3 points would provide moderate-strength evidence for pathogenicity, defined as a “Pathogenic moderate (PM7)” evidence level. However, no similar framework has been suggested for *CALM* gene mutations. Therefore, we can just propose that the p.E140Q mutation is likely pathogenic. Further functional studies are warranted.

In case 2, the mutation has been previously reported in multiple countries and regions, including Japan, France, the United Kingdom, and Spain, mostly as *de novo* mutations in independent families (11–16). Although the clinical phenotypes vary, presenting as CPVT, LQTS, or an overlap phenotype, the core features are highly consistent, including early age of onset (median 4.5, range 2–7 years), higher incidence in males, more severe phenotypes in males (17), induction by exercise or emotional stress, and polymorphic ventricular arrhythmias. Most cases respond well to β-blocker monotherapy and maintain long term event free survival including those with overlap phenotype. However, as shown by the large-sample data from the International Calmodulinopathy Registry (4), the efficacy of β-blocker monotherapy is significantly limited in patients with calmodulinopathy. More than half of patients experience recurrent cardiac events during medical therapy, and β-blocker monotherapy fails to provide adequate survival protection. In the present case, syncope still occurred despite initial propranolol monotherapy and subsequent combined therapy with propranolol and mexiletine, and the QTc interval remained prolonged, indicating treatment failure. The negative lidocaine test indicates a poor response to mexiletine, further suggesting that in such patients, strategies relying on sodium channel blockers to correct the QTc interval are often ineffective, and treatment should focus more on suppressing adrenergic triggers. Furthermore, this patient exhibited significant bradycardia. A previously reported male CPVT patient carrying the p.N98S mutation, who died suddenly without intervention, also displayed marked sinus bradycardia on ECG (12), suggesting that bradycardia may predict more severe disease phenotypes (4, 18). Therefore, in individuals with recurrent arrhythmic syncope and significant sinus bradycardia, evaluation for ICD therapy may be warranted, given the high risk of malignant ventricular arrhythmias and conduction system involvement in this phenotype. In summary, compared with most previously reported p.N98S cases, our patient shares the core phenotypic features (such as early age of onset at 4 years, male sex, exercise- or emotion-induced polymorphic ventricular arrhythmias) but shows notable differences in treatment response (more refractory) and bradycardia, which may indicate a more severe phenotype.

Previous reports have revealed that patients with the LQTS/CPVT overlap phenotype account for approximately 20%–30% of calmodulinopathies, and their clinical characteristics differ from those of patients with “pure LQTS”. Patients with the overlap phenotype are diagnosed at a relatively older age (mean 7 years vs. 14 months), have a shorter QTc interval (mean 0.519 s vs. 0.622 s), and have a lower incidence of cardiac arrest (33% vs. 86%) (8). The two pediatric patients in this report were diagnosed at ages 13 and 15 years, with initial QTc intervals of 0.51–0.55 s, and no cardiac arrest were documented, which is consistent with the characteristics of the overlap phenotype reported in the literature.

Of note, calmodulinopathies are often misdiagnosed as “genotype-negative LQTS” or “epilepsy”. Even when a CaM mutation is identified, approximately 20% of the patients with a CPVT phenotype are often initially misdiagnosed as LQTS, because of the sole detection of QTc prolongation without identification of the ventricular arrhythmias that occur exclusively during exercise and stress (19). The importance of this identification lies in the fact that comprehensive therapy tailored to the phenotypes is crucial for controlling arrhythmias. Both two cases in our report were initially diagnosed as LQTS associated with CaM mutations before their admission since the resting ECG showed no other abnormalities except bradycardia and QTc prolongation. The concealed CPVT phenotypes were ultimately unmasked by exercise challenge ECG. Therefore, when hereditary malignant arrhythmias are suspected, gene testing can assist in achieving a more accurate diagnosis. Furthermore, for patients with CaM gene mutations and QTc prolongation, using exercise testing to distinguish whether the clinical phenotype is isolated LQTS or LQTS/CPVT overlap phenotype is crucial for precise treatment, especially in patients presenting with exercise- or stress-induced syncope.

Regarding treatment, patients with calmodulinopathy-associated LQTS/CPVT overlap phenotype require consideration of both arrhythmic mechanisms, with β-blockers as the cornerstone (4, 20). The addition of a Class Ic agent may further reduce ventricular arrhythmias, as Class Ic drugs (e.g., flecainide, propafenone) directly inhibit the RyR2 channel via open-state block, thereby reducing calcium-induced calcium release and triggered activity, whereas class Ib agents (e.g., mexiletine) lack this effect (21, 22). In this report, case 1 was promptly diagnosed with an LQTS/CPVT overlap phenotype and was treated with propranolol combined with propafenone, achieving satisfactory therapeutic outcomes. Case 2 experienced syncope recurrence and progressive QTc prolongation to 0.62 s despite receiving propranolol combined with mexiletine, a drug that is typically used in treating LQTS. Short-term efficacy awaits observation after switching mexiletine to propafenone, but the favorable response in case 1 provides a clinical rationale for further exploration. Moreover, for patients who have experienced cardiac arrest or are refractory to medical therapy, ICD implantation should be considered (20); and patients with recurrent syncope and sinus bradycardia may also need to be considered on a case-by-case basis (4, 18). However, it is important to note that ICD shocks in patients with CPVT may trigger catecholamine release, potentially leading to electrical storm. Therefore, ICD programming should be carefully configured to delay delivery of shocks whenever possible (23). Currently, targeted therapy for calmodulinopathies is a research hotspot. Antisense oligonucleotide therapy has achieved proof-of-concept success in animal models (24), and gene editing approaches as well as “suppression-replacement” gene therapy are being actively explored (25).

Strengths of this report include longitudinal follow-up from age 7 to 15 with precise dose documentation and adherence assessment, confirming the pharmacoresistant trajectory, and the identification of *de novo CALM2* mutations enabling genotype–phenotype comparison.

Limitations include the small sample size precluding generalization, the absence of functional studies to characterize variant-specific effects, the unclear rationale for the marked dose reduction, the family's declination of ICD implantation, and the lack of deep-sequencing to exclude low-level parental germline mosaicism (26), which together limit conclusions about true drug efficacy and optimal salvage strategies. Additionally, the patient perspective on treatment burden was not obtained. Nonetheless, these cases provide real-world evidence supporting consideration of Class Ic agents and early ICD implantation in refractory *CALM2*-associated overlap phenotype.

## 4 Conclusion

We report LQTS/CPVT overlap phenotype caused by *CALM2* p.E140Q and p.N98S mutations in Chinese children with calmodulinopathy. For such patients, exercise stress testing and genetic testing are helpful for establishing a definitive diagnosis and guiding treatment. A combination of β-blockers and Class Ic antiarrhythmic agents should be employed and ICD implantation should be considered when necessary. In the future, gene-targeted therapy for calmodulinopathies is expected to overcome the limitations of current treatment approaches, facilitating a transition from empirical therapy to precision medicine.

## Data Availability

The datasets presented in this article are not readily available due to ethical and privacy considerations. Requests to access the datasets should be directed to the corresponding author.
